# Beyond the Jump: A Scoping Review of External Training Load Metrics in Volleyball

**DOI:** 10.1177/19417381241237738

**Published:** 2024-03-31

**Authors:** André Rebelo, João R. Pereira, Fábio Y. Nakamura, João Valente-dos-Santos

**Affiliations:** † CIDEFES, Centro de Investigação em Desporto, Educação Física e Exercício e Saúde, Universidade Lusófona, Lisboa, Portugal; ‡ COD, Center of Sports Optimization, Sporting Clube de Portugal, Lisbon, Portugal; § Research Center in Sports Sciences, Health Sciences and Human Development (CIDESD), University Institute of Maia (ISMAI), Maia, Portugal

**Keywords:** athletic performance, measurement technologies, movement planes, multidimensional analysis, player positions

## Abstract

**Context:**

Volleyball is a complex sport involving multifaceted movements and high-velocity actions, leading to diverse external training loads (ETLs) that have profound implications for player performance and injury risk.

**Objective:**

To provide a comprehensive overview of the measurement of ETL in volleyball, identify gaps in current understanding, and offer valuable insights for stakeholders in the field.

**Data Sources:**

The literature search was conducted across the following electronic databases: PubMed/Medline, Scopus, Web of Science, and SPORTDiscus.

**Study Selection:**

Studies were selected based on their relevance to the measurement of ETL in volleyball.

**Study Design:**

A scoping review methodology was chosen to map and summarize the broad body of literature related to ETL measurement in volleyball.

**Level of Evidence:**

Level 4.

**Data Extraction:**

Data related to ETL measurements in volleyball were extracted and analyzed from the selected studies, focusing on metrics utilized, player positions examined, and technologies employed.

**Results:**

A total of 18 studies related to ETL in volleyball were identified and examined for this review. Despite the importance of sagittal plane movements in volleyball, the review identified a substantial research gap regarding ETL measurements beyond this plane, as well as a lack of focus on the unique demands of different player positions like the liberos. The use of technologies such as inertial measurement units was prevalent, but more comprehensive measurement methods are needed.

**Conclusion:**

There is a critical need for diversified ETL metrics in volleyball, extending beyond the conventional sagittal plane measurements. The findings highlight a substantial research gap in addressing the unique demands of different player positions, notably the liberos. This study underscores the importance of incorporating multiplanar movement data, player-specific roles, and advanced measurement technologies to develop more tailored training programs and injury prevention strategies.

Volleyball is a sport rich in multifaceted movements, strategic planning, and high-velocity actions, leading to varied and often intense external training loads (ETLs).^10,41^ ETLs, encompassing the volume, intensity, and type of physical work performed by an athlete during training or competition, have profound implications for player performance, injury risk, and recovery needs.^17^ These loads are especially variable in volleyball due to the sport’s inherent dynamic nature, marked by unpredictable gameplay and player-specific roles.^41,42^

These roles, such as that of the libero, highlight the complexity and unique demands of volleyball. The libero, known for their defensive mastery, often engages in quick, low-to-ground movements, requiring rapid acceleration, deceleration, and changes in direction.^12^ Unlike other players who frequently jump for attacks or blocks, the libero’s performance involves predominantly lateral and diagonal maneuvers, pointing to a unique ETL profile. Middle blockers also need to move laterally along the net to block attacks from different zones of the court, thereby involving significant transverse and frontal plane actions.^2,26^ Like liberos, outside hitters also perform lateral and diagonal movements during defence, especially when transitioning from backcourt to the attack line.^22,28^ In addition, the rotational movements of outside hitters while spiking can generate significant torsional loads on the body, emphasizing the necessity of a triaxial ETL measurement.^8^ Setters frequently perform rapid changes of direction, and their hands-on role in ball distribution involves substantial rotations, highlighting the relevance of the transverse plane.^31^ Such positional nuances accentuate the need for a thorough, multidimensional understanding of ETL in volleyball.

Advanced technologies like accelerometers, inertial measurement units (IMUs), and global positioning system (GPS) devices have revolutionized the way ETL is quantified and interpreted.^13,35^ Despite these technological advances, the focus of ETL measurement in volleyball has been skewed toward movements in the sagittal plane, such as jump count and jump height.^25^ This approach overlooks the vast range of motions that volleyball players, particularly liberos, undertake on the court.

In parallel, there is a growing body of research exploring the internal training load - the individual physiological and psychological response to ETL.^17^ Although these studies have provided valuable insights into players’ loading patterns,^37^ the need to complement this understanding with a comprehensive analysis of ETL, extending beyond sagittal plane movements, is becoming increasingly apparent. Given the fast-paced, demanding, and position-specific nature of volleyball, gaining a holistic perspective on ETL is crucial. This involves not only exploring the range of ETL metrics used across different studies, but also identifying the measurement tools and technologies employed and the player positions and levels of play considered in the research. Such a comprehensive overview would not only enhance our understanding of volleyball’s physical demands but also assist in developing tailored training programs and injury prevention strategies. This can be important in recognizing the intensity and volume of training that is most effective for enhancing specific skills or physical attributes. This means that training can be aligned more precisely with the demands of specific playing positions or individual weaknesses and strengths, including in volleyball.^24^ Furthermore, through ongoing assessment of ETL, coaches can identify signs of fatigue or overtraining, allowing them to adjust training loads and incorporate appropriate rest and recovery protocols.^14,15^ This ensures that athletes remain fresh and responsive to training stimuli. By understanding the specific physical demands during games, training programs can be designed to mimic these demands, ensuring that players are conditioned to meet the specific challenges they will face in matches.

Given the exploratory nature of the present research, a scoping review was deemed an ideal approach to gather and present the breadth of information related to ETL measurement in volleyball.^46^ The objective of this review was therefore to provide a clear and nuanced overview of the measurement of ETL in volleyball, to identify gaps in the current understanding, and to offer valuable insights for researchers, practitioners, and stakeholders in the field.

## Methods

### Protocol and Registration

This review protocol was conducted and reported in accordance with the Preferred Reporting Items for Systematic Reviews and Meta-Analyses Extension for Scoping Reviews (PRISMA-ScR) guidelines.^46^ All aspects of the PRISMA-ScR checklist are available in the Appendix (available in the online version of this article). The scoping review protocol was developed and registered on the International Platform of Registered Systematic Review and Meta-analysis Protocols (INPLASY).^34^ The protocol was compiled to provide comprehensive information and ensure transparency and replicability of the planned research. Methodological decisions, inclusion and exclusion criteria, the search strategy, and the approach to data extraction and synthesis were all outlined in this protocol.

To ensure accessibility and transparency, the protocol has been made publicly available on the INPLASY platform. It was published on June 19, 2023, and can be accessed via the direct link: https://inplasy.com/inplasy-2023-6-0059/. Its unique digital object identifier (DOI) is 10.37766/inplasy2023.6.0059, and the INPLASY registration number is INPLASY202360059.

### Eligibility Criteria

Studies were included based on the following criteria: (1) the study involves volleyball players of any age and performance level; (2) the study must report on ETL, how it is measured, and ideally, how it relates to player performance, injury rates, or other relevant outcomes; (3) the study must report on ETL measurements that extend beyond movements in the sagittal plane or clearly state that only sagittal plane movements are considered; (4) both observational and interventional studies were included.

These criteria are selected to capture a broad range of studies that examine ETL in volleyball. By including players of all ages and performance levels, the review can provide a comprehensive understanding of ETL measurement across diverse populations.

Restrictions were applied based on the language of publication. Only studies published in English were considered to ensure that the research team could accurately interpret and synthesize the findings. There were no restrictions on the date of publication to ensure a comprehensive overview of all relevant research.

### Information Sources

The literature search was conducted across the following electronic databases: PubMed/Medline, Scopus, Web of Science, and SPORTDiscus. The date of the last literature search was June 26, 2023. In addition to the database searches, reference lists of identified articles and relevant reviews were manually scanned for additional studies. Similarly, key journals in the field were hand-searched for relevant articles.

### Search

The search strategy was developed and executed by the primary researcher, with input from the entire research team. The strategy conducted was in accordance with the Peer Review of Electronic Search Strategies (PRESS) checklist to ensure its robustness and comprehensiveness.^4^

The search strategy encompasses both controlled vocabulary (such as MeSH terms in PubMed/Medline) and free-text terms to capture all relevant literature. The key search terms were “volleyball,” “external training load,” “physical demands,” “measurement,” and “libero,” along with their synonyms, related terms, and any appropriate variations. To ensure thoroughness and reproducibility, the search strategy was adapted to the syntax and subject headings of each database. For instance, in PubMed/Medline, the search looked like this: (“volleyball”[MeSH Terms] OR “volleyball”[All Fields]) AND (“training load”[All Fields] OR “physical demands”[All Fields]) AND “measurement”[All Fields]. The comprehensive search terms and strategy for each database are reported in the Appendix (available in the online version of this article) to allow for easy replication.

### Selection of Sources of Evidence

The selection process for included sources of evidence was undertaken in several steps. Initially, after executing the search strategy across the electronic databases, all identified citations were imported into the CADIMA software. This platform was chosen for its ability to facilitate the screening and data extraction processes and to ensure a structured and systematic approach to the review.^18^ It also facilitated the removal of duplicate citations, which was the first step in the screening process.

Subsequently, a standardized form was developed to guide the screening of titles and abstracts. This form included questions related to the eligibility criteria, such as whether the study involved volleyball players, whether it reported on ETL, and whether it reported measurements beyond the sagittal plane. The form was tested by the 4 authors of the review on a small subset of 10 citations. This calibration exercise helped ensure that all authors had a clear understanding of the eligibility criteria and could apply them consistently. Discrepancies were discussed and resolved through consensus, leading to a refinement of the form and a clearer shared understanding of the eligibility criteria.

After the calibration exercise, each title and abstract was screened by 2 authors, with conflicts resolved by a third author. This double screening approach was adopted to minimize the risk of bias and to ensure no relevant studies were missed. A similar approach was used for the full-text screening of articles identified as potentially eligible during the title and abstract screening stage. For the full-text screening, a more detailed form was used, capturing information related to study design, participant characteristics, and outcome measures.

In the event of disagreements that could not be resolved by discussion, a third author acted as a mediator to achieve consensus. The CADIMA software facilitated the management of this process, including tracking which authors screened each article and recording decisions about study inclusion and reasons for exclusion.

### Data Charting

A data charting form was developed to standardize the extraction of relevant information from each included study. This form was designed to capture a range of information pertinent to the research question, including study characteristics (such as author, year, and study design), participant characteristics (such as age, performance level, and player position), and details about ETL measurement (including the specific metrics used, any measurement tools or technologies, and key findings).

The selection of items for charting was informed by the research question and the principles of a scoping review. To ensure consistency and reliability in data charting, the team carried out a calibration exercise, similar to the one performed during the study selection process. A subset of 5 included studies was used for this exercise. Each author independently charted the data from these studies, and then the team compared and discussed their charting to identify and resolve discrepancies.

Once the charting form was finalized, the data extraction process was initiated. Each included study was independently charted by 2 authors using the CADIMA software. This tool was particularly useful in managing the extraction process and facilitating collaboration among the team. When inconsistencies arose, these were discussed and resolved through consensus, with the involvement of a third author if required. To further ensure accuracy, a 10% random sample of the charted data was verified by a third author, checking for any errors or omissions in the data extracted.

During the charting process, the team maintained an iterative approach, revising the charting form as necessary. These changes were based primarily on emerging insights from the data and were made to improve the clarity and relevance of the extracted information. If necessary, authors of the original studies were contacted for clarification or additional information to ensure accurate data extraction.

### Data Items

In this scoping review, the data charting form was designed to extract a combination of qualitative and quantitative information from each of the included studies. We began by recording study identification details including the author(s), the year of publication, and the country where the study was conducted. We also captured information about the study design, detailing the methodology used, such as whether it was a cross-sectional, longitudinal, case study, or another design.

The characteristics of the participants in each study were also documented, with information regarding age, sex, level of play (which could range from youth/high school to collegiate to professional), and their specific player position. To understand the metrics used in the studies, we extracted specific ETL metrics, which could include total distance covered, number of accelerations or decelerations, number of jumps, among others. The measurement tools or technologies used to gather these metrics were also recorded, and these could range from local positioning systems (LPSs) to video analysis or other wearable devices.

An important part of the charting process was extracting key findings of each study related to the measurement of ETL in volleyball players. The findings, along with any limitations or challenges reported by the authors in relation to measuring ETL, were synthesized into concise statements.

Each item in the data charting form was clearly defined to ensure a consistent understanding and application among all authors. The systematic capture of this wide range of information facilitated a comprehensive understanding of the existing body of evidence regarding the measurement of ETL in volleyball players, thereby highlighting the current gaps and potential areas for future research.

### Critical Appraisal of Individual Sources of Evidence

Given that the objective of this scoping review is to identify and map the approaches used to measure ETL in volleyball players, a critical appraisal step may not be necessary.^46^ The main goal is to provide an overview of the existing research and highlight gaps and opportunities for further investigation. Therefore, the present scoping review did not include a formal critical appraisal of the individual sources of evidence.

### Synthesis of Results

The synthesis of the results aimed to create a comprehensive overview of the range of evidence included in this review, aligned with the research questions and objectives. To facilitate this, the extracted data were processed in a structured manner to generate a narrative synthesis. This process involved an iterative exploration of the data, identification of patterns and themes, and generation of insights relevant to the measurement of ETL in volleyball players.

The synthesis of results took primarily a descriptive approach, characterizing the overall body of literature and detailing the key findings across studies. This included information on the study designs employed, the characteristics of the participant populations, the ETL metrics reported, the measurement tools and technologies used, and the key findings of each study. This allowed the current review to provide a comprehensive and nuanced picture of the research landscape.

The findings from the synthesis are presented in several ways to cater to diverse information needs and to enhance accessibility and understanding of the results. A narrative summary is provided, detailing the key insights and trends across the studies. In addition, the data are presented visually using tables to illustrate the range and distribution of the studies, the participant characteristics, the ETL metrics used, and other relevant aspects. These visual representations complemented the narrative synthesis, providing an at-a-glance understanding of the key aspects of the evidence base.

## Results

### Selection of Sources of Evidence

**Figure 1.**
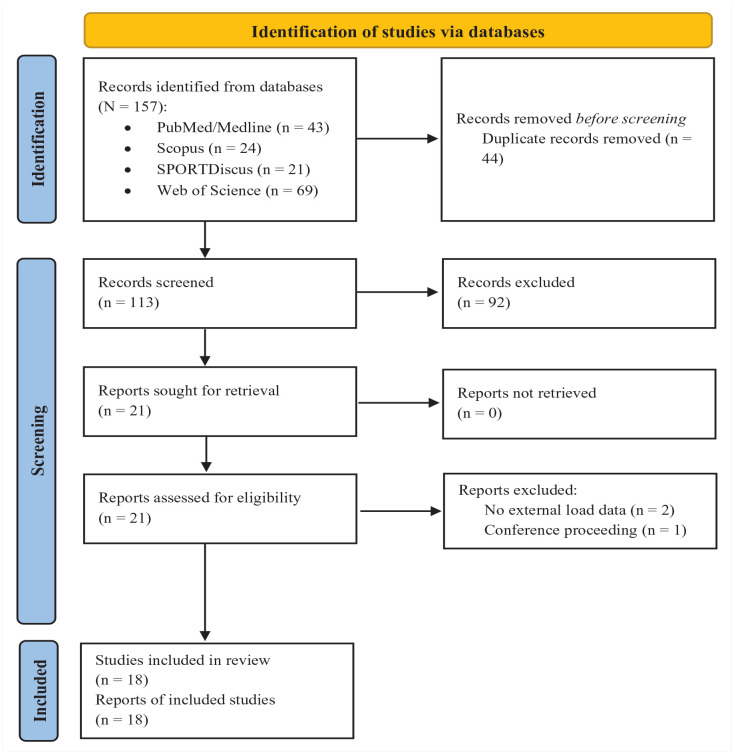
PRISMA 2020 flow diagram for the present review.

The search for sources of evidence in the literature resulted in the identification of 157 records across 4 major databases: PubMed/Medline (43 records), Scopus (24 records), SPORTDiscus (21 records), and Web of Science (69 records). Before initiating the screening process, 44 duplicate records were meticulously identified and eliminated, reducing the total number of unique records to 113. After the removal of duplicates, all 113 records were screened thoroughly for relevance to the research objectives. This initial screening process led to the exclusion of 92 records, due primarily to reasons such as mismatch with the study focus, lack of pertinent data, or studies not focused on volleyball players. The remaining 21 records were then earmarked for more detailed evaluation. These records were retrieved for a more in-depth review and assessment against the inclusion and exclusion criteria. Of these, 3 records were excluded: 2 were identified as not having any ETL data, and 1 was a conference proceeding which did not meet the inclusion criteria. Ultimately, 18 studies met all the stipulated criteria and were included in the final scoping review. The process of record identification, screening, eligibility, and inclusion in the study is presented in a flow diagram in Figure 1.

### Characteristics of Sources of Evidence

The 18 studies included in this review present a range of participant characteristics, reflecting diverse contexts of volleyball training and performance. The studies varied in terms of sample size, ranging from a minimum of 5 participants to a maximum of 24 participants.^21,29,40^ The participants represented both male and female volleyball players. ^1,6,7,11,16,19,20,21,23,25,27,29,32,33,36,40,42,43^ Age ranges were also quite diverse across studies, with the youngest participants being 16 to 18 years old and the oldest participants up to 30.5 years old.^25,32^ In terms of player positions, most of the studies reported on outside hitters, setters, middle blockers, and opposites (n = 7; 39%).^11,20,23,25,33,36,42^ A smaller number of studies also reported on liberos (n = 4; 22%).^6,19,27,43^ The level of play was predominantly at the elite level (n = 12; 67%),^1,6,7,11,20,21,23,25,27,33,36,42^ but there were also studies involving collegiate (n = 4; 22%),^16,19,29,43^ and youth (n = 2; 11%) players.^32,40^ In terms of physical characteristics, the studies reporting these data showed variations in height and weight across different player positions and levels of play. However, some studies did not report these data (n = 4; 22%).^16,33,40,42^

Further details about the individual studies, including specific participant characteristics, level of play, and player positions, are provided in Table 1. These findings present a broad overview of the populations involved in research on the measurement of ETL in volleyball, highlighting the variability across studies and the diverse contexts in which this research has been conducted.

**Table 1.** Descriptive data of the included studies

Study	N	Sex	Age, y	Height, cm	Weight, kg	Level	Player Positions
Lima et al, 2023^25^	7	Male	30.5 ± 3.5	200.2 ± 6.3	93.0 ± 8.1	Elite	Outside hitter, setter, middle blocker, and opposite
Schumann et al, 2023^40^	24	Female	Not reported	Not reported	Not reported	Youth	Not reported
Rebelo et al, 2023^38^	15	Male	28.51 ± 5.39	193.19 ± 9.87	88.46 ± 13.18	Elite	Outside hitter, setter, middle blocker, and opposite
Taylor et al, 2022^43^	16	Female	19.6 ± 1.1	178 ± 7.0	75.9 ± 9.3	Collegiate	Libero, outside hitter, setter, middle blocker, and opposite
Piatti et al, 2022^33^	12	Male	25.8 ± 2.37	201.1 ± 0.05	Not reported	Elite	Outside hitter, setter, middle blocker, and opposite
de Leeuw et al, 2022^6^	17	Male	27.0 ± 3.0	200 ± 10.0	91.2 ± 6.4	Elite	Libero, outside hitter, setter, middle blocker, and opposite
Herring and Fukuda, 2022^16^	9	Female	Not reported	Not reported	Not reported	Collegiate	Outside hitter, middle blocker, and opposite
Altundag et al, 2022^1^	14	Female	22 ± 0.9	195.1 ± 7.6	71.4 ± 6.3	Elite	Not reported
Lima et al, 2022^23^	14	Male	21.7 ± 4.19	192.4 ± 6.25	85.7 ± 8.69	Elite	Outside hitter, setter, middle blocker, and opposite
Pawlik and Mroczek, 2022^32^	11	Female	16-18	172.42 ± 7.3	63.54 ± 8.36	Youth	Not reported
de Leeuw et al, 2022^7^	14	Male	27 ± 3	197 ± 7.0	90.5 ± 6.3	Elite	Not reported
Lima et al, 2021^20^	10	Male	21.7 ± 4.19	192.4 ± 6.25	85.7 ± 8.69	Elite	Outside hitter, setter, middle blocker, and opposite
Kupperman et al, 2021^19^	11	Female	19.36 ± 1.27	64.04 ± 7.08	69.91 ± 3.88	Collegiate	Libero, outside hitter, setter, middle blocker, and opposite
García-de-Alcaraz et al, 2020^11^	11	Male	28.0 ± 6.12	197.2 ± 6.93	87.9 ± 6.41	Elite	Outside hitter, setter, middle blocker, and opposite
Ness et al, 2019^29^	5	Female	19.6 ± 1.1	180 ± 1.0	79.4 ± 6.6	Collegiate	Not reported
Maciel Rabello et al, 2019^27^	18	Male	23.0 ± 4.3	190 ± 0.0	89.8 ± 9.6	Elite	Libero, outside hitter, setter, middle blocker, and opposite
Lima et al, 2019^20^	5	Male	26.7 ± 7.2	193 ± 8.0	86.5 ± 6.3	Elite	Outside hitter, setter, middle blocker
Skazalski et al, 2018^42^	14	Male	Not reported	Not reported	Not reported	Elite	Outside hitter, setter, middle blocker, and opposite

### Results of Individual Sources of Evidence

The 18 studies reviewed utilized varying methods and technologies for measuring ETLs in volleyball, with durations ranging from short-term, 5-day studies to long-term studies spanning over 2 full competitive seasons.^16,32^

The dominant technology used across studies was the IMU equipped with 3-axis gyroscopes, magnetometers, and accelerometers.^6,7,20,21,23,25,33,36,40,42,43^ This device was commonly placed on a belt secured to the athletes’ hips, transmitting data via Bluetooth. The validity of the IMU device was typically reported according to the work of Charlton et al,^5^ specifically the Vert Classic model (MyVert). Notably, Pawlik and Mroczek^32^ utilized the ClearSky Vector S7 (Catapult Innovations), while Kupperman et al^19^ used the ClearSky T6 (Catapult Innovations). In a departure from the IMU method, Altundag et al^1^ used an LPS (KINEXON, GMBH, Precision Technologies, KINEXON ONE), and the validity of this method was reported according to Fleureau et al.^9^ Both García-de-Alcaraz et al^11^ and Ness et al^29^ utilized video camera methods, and Maciel Rabello et al^27^ used a combination of 3-axis accelerometers and video camera to measure the ETL. The methodological considerations varied across the studies, with many describing the placement of the device, the rate of data sampling, or specific details about how data were collected or processed.

In terms of the training phase during which these studies were conducted, a large number of the studies collected data during both the preparatory (pre-season) and competitive (in-season) phases (n = 9; 50%),^1,6,7,11,19,23,29,40,42,43^ while others focused solely on the competitive phase (n = 7; 39%).^16,20,21,25,32,33,36^

Detailed information regarding the results of the individual sources of evidence is in Table 2. The collective evidence reflects a strong interest in the use of IMU technology to measure ETL in volleyball, with a specific preference for devices equipped with 3-axis gyroscopes, magnetometers, and accelerometers.

**Table 2.** Study settings and methodological considerations of the included studies

Study	Duration of the Study	Training Phase	Type of Device(s)	Validity and Reliability of Device Reported	Methodological Considerations
Lima et al, 2023^25^	3 weeks	Competitive (in-season)	IMU with 3-axis gyroscopes, 3-axis magnetometers, and 3-axis accelerometers (Vert Classic, MyVert)	Validity reported according to Charlton et al^5^	IMU placed on a belt secured to athletes’ hips. Data transmitted via Bluetooth
Schumann et al, 2023^40^	20 weeks	Preparatory (pre-season) and competitive (in-season)	IMU with 3-axis gyroscopes, 3-axis magnetometers, and 3-axis accelerometers (Vert Classic, MyVert)	Validity reported according to Benson et al^3^	IMU placed on a belt secured to athletes’ hips. Data transmitted via Bluetooth
Rebelo et al, 2023^38^	5 weeks	Competitive (in-season)	IMU with 3-axis gyroscopes, 3-axis magnetometers, and 3-axis accelerometers (Vert Classic, MyVert)	Validity reported according to Charlton et al^5^	IMU placed on a belt secured to athletes’ hips. Data transmitted via Bluetooth
Taylor et al, 2022^43^	17 weeks	Preparatory (pre-season) and competitive (in-season)	IMU with 3-axis gyroscopes, 3-axis magnetometers, and 3-axis accelerometers (Vert Classic, MyVert)	Validity reported according to Charlton et al^5^	IMU placed on a belt secured to athletes’ hips. Data transmitted via Bluetooth
Piatti et al, 2022^33^	1 competitive season	Competitive (in-season)	IMU with 3-axis gyroscopes, 3-axis magnetometers, and 3-axis accelerometers (Ver Classic, MyVert)	Validity reported according to Charlton et al^5^	IMU placed on a belt secured to athletes’ hips. Data transmitted via Bluetooth
de Leeuw et al, 2022^7^	24 weeks	Preparatory (pre-season) and competitive (in-season)	IMU with 3-axis gyroscopes, 3-axis magnetometers, and 3-axis accelerometers (Vert Classic, MyVert)	Validity reported according to Charlton et al^5^	IMU placed on a belt secured to athletes’ hips. Data transmitted via Bluetooth
Herring and Fukuda, 2022^16^	2 competitive seasons	Competitive (in-season)	IMU with 3-axis gyroscopes, 3-axis magnetometers, and 3-axis accelerometers (Vert Classic, MyVert)	Validity reported according to Charlton et al^5^	IMU placed on a belt secured to athletes’ hips. Data transmitted via Bluetooth
Altundag et al, 2022^1^	10 months	Preparatory (pre-season) and competitive (in-season)	Local positioning system (KINEXON, GMBH, Precision Technologies, KINEXON ONE)	Validity reported according to Fleureau et al^9^	External load data collected at 20 Hz and processed using specific Kinexon software. The system used in this study consisted of 12 antennae positioned at 3 different distances around the volleyball court. The data acquisition sensor was placed in the middle of each athlete’s upper back using a vest. Signals in the frequency range of 4.25-7.25 GHz were transmitted to antennas using UWB technology
Lima et al, 2022^23^	8 months	Preparatory (pre-season) and competitive (in-season)	IMU with 3-axis gyroscopes, 3-axis magnetometers, and 3-axis accelerometers (Vert Classic, MyVert)	Validity reported according to Charlton et al^5^	IMU placed on a belt secured to athletes’ hips. Data transmitted via Bluetooth
Pawlik and Mroczek, 2022^32^	5 days	Competitive (in-season)	IMU with 3-axis gyroscopes, 3-axis magnetometers, and 3-axis accelerometers (ClearSky Vector S7; Catapult Innovations)	Not reported	Accelerometer sampled at 1 kHz, provided at 100 Hz
de Leeuw et al, 2022^6^	24 weeks	Preparatory (pre-season) and competitive (in-season)	IMU with 3-axis gyroscopes, 3-axis magnetometers, and 3-axis accelerometers (Vert Classic, MyVert)	Validity reported according to Charlton et al^5^	IMU placed on a belt secured to athletes’ hips. Data transmitted via Bluetooth
Lima et al, 2021^20^	10 weeks	Competitive (in-season)	IMU with 3-axis gyroscopes, 3-axis magnetometers, and 3-axis accelerometers (Vert Classic, MyVert)	Validity reported according to Charlton et al^5^	IMU placed on a belt secured to athletes’ hips. Data transmitted via Bluetooth
Kupperman et al, 2021^19^	18 weeks	Preparatory (pre-season) and competitive (in-season)	IMU with 3-axis gyroscopes, 3-axis magnetometers, and 3-axis accelerometers (ClearSky T6; Catapult Innovations)	Reliability reported according to Nicolella et al^30^	Units placed on the back of players (between the scapulae) in a pocket sewn into a fitted Catapult Sports harness. Units sampled at a rate of 100 Hz
García-de-Alcaraz et al, 2020^11^	32 weeks	Preparatory (pre-season) and competitive (in-season)	Video camera (Sony Handycam HDR-CX240)	Not reported	A Sony Handycam HDR-CX240 (30 Hz) video camera, located at the end of the court and at a height greater than the official volleyball net height, was used to record all in-court training sessions
Ness et al, 2019^29^	22 weeks	Preparatory (pre-season) and competitive (in-season)	Video camera	Not reported	Practices were viewed by 2 researchers collaboratively in an environment with minimal distractions. An upper extremity swing was tallied for a serve, attack, or roll shot. One researcher viewed the video footage and called out a swing along with the corresponding athlete’s jersey number, while the other researcher recorded the data
Maciel Rabello et al, 2019^27^	7 weeks	Preparatory (pre-season)	3-axis accelerometers (Zephyr BioHarness 3TM, Zephyr Technology) and video camera	Not reported	Jumps were counted based on pattern recognition in the vertical acceleration data (100 Hz)
Lima et al, 2019^24^	4 weeks	Competitive (in-season)	IMU with 3-axis gyroscopes, 3-axis magnetometers, and 3-axis accelerometers (Vert Classic, MyVert)	Validity reported according to Charlton et al^5^	IMU placed on a belt secured to athletes’ hips. Data transmitted via Bluetooth
Skazalski et al, 2018^42^	1 full season	Preparatory (pre-season) and competitive (in-season)	IMU with 3-axis gyroscopes, 3-axis magnetometers, and 3-axis accelerometers (Vert Classic, MyVert)	Validity reported according to Charlton et al^5^	IMU placed on a belt secured to athletes’ hips. Data transmitted via Bluetooth

IMU, inertial measurement unit; UWB, ultrawide band.

### Synthesis of Results

According to the data presented in Table 3, the majority of studies focused on variables measured in the sagittal plane, specifically relating to jumps (n = 17; 94%). Variables included the count, height (including maximum, minimum, average, and range), and load of jumps, in addition to factors such as the jump rate and height >50 cm. Lima et al^25^ highlighted the role of jump count, maximum jump height, and average jump height, finding that a higher number of jumps corresponded to greater jump height. Meanwhile, Piatti et al^33^ highlighted the specific case of middle blockers, who presented the highest jump load and second highest number of jumps, despite not jumping as frequently as setters. Taylor et al^43^ tied these variables to injury, noting that injured athletes performed fewer jumps and had larger variation in ETLs before their injury.

**Table 3.** Variables measured and main results of the included studies

Study	Variables Measured	Main Results
Lima et al, 2023^25^	Sagittal plane: jump count, maximum jump height, and average jump height	A similar density of jumps was observed during the week. However, when comparing MD-1 to MD-2, a more significant average number of jumps per minute was observed in MD-1. In addition, a positive, large, and significant correlation was registered between the number of jumps and the height of the jump
Schumann et al, 2023^40^	Sagittal plane: jump count, jumps >50 cm, maximum jump height, and stress upon landing. Frontal and transverse planes: movements per minute All planes: kinetic energy	The club elite sample of players had greater weekly variations throughout the season and many weeks were outside of the optimal ACWR range. The collegiate data showed spikes in ACWR at the onset of the season
Rebelo et al, 2023^38^	Sagittal plane: jump count, average jump height, and kinetic energy	Sleep quality and fatigue were associated negatively with weekly kinetic energy
Taylor et al, 2022^43^	Sagittal plane: jump count, average jump height, maximum jump height, and jump load	Those athletes who were injured performed significantly fewer jumps per athletic exposure and had larger variation in external training loads before their injury
Piatti et al, 2022^33^	Sagittal plane: jump count and jump height	Middle blockers present the highest jump load; they have a median number of jumps of 126 (second only to the 147 of setters) and have the highest number of jumps >50 cm. Setters present the highest number of jumps per game and the lowest jump height
de Leeuw et al, 2022^6^	Sagittal plane: jump count, and average jump height	Worse attack performance is linked to low jump heights and small variations in the number of high jumps in the 4 weeks before competition. Lower passing performance was associated with small variations in the number of high jumps in the preceding week and an excessive number of high jumps performed, on average, in the 2 weeks before competition
Herring and Fukuda, 2022^16^	Sagittal plane: jump count, jump rate, and average jump height	Outside hitters had the highest average jump height followed by middle blockers then opposites. Middle blockers had higher jump count and jump rate compared with outside hitters
Altundag et al, 2022^1^	Sagittal plane: jump count. Frontal and transverse plane: total distance, accelerations, decelerations, metabolic load, and speed	As the metabolic load, jumps, accelerations, and decelerations values obtained during training increase, the values obtained in competitions also increase
Lima et al, 2022^23^	Sagittal plane: jump count, minimum jump height, maximal jump height, range jump height, and average jump height	There was a negative correlation between the maximum jump height and the range jump height with the internal training load. Higher internal intensities are correlated with lower external intensities in sessions further away from the game day
Pawlik and Mroczek, 2022^32^	Sagittal plane: jump count and jump height. Frontal and transverse planes: accelerations and decelerations All planes: Player Load	There is a relationship between internal training load and the number of total accelerations. It was reported that on the training days of the same well-being level, the jump number values were significantly different
de Leeuw et al, 2022^7^	Sagittal plane: jump count and jump height	Jump load is an important predictor for overuse injuries in volleyball
Lima et al, 2021^20^	Sagittal plane: jump count and jump height	Congested features did not interfere with the players’ external training load
Kupperman et al, 2021^19^	Sagittal plane: jump count and jump height. Frontal and transverse planes: changes of direction, repeated high-intensity efforts, accelerations, and decelerations All planes: Player Load	Setters accrued over twice as many jumps in a practice than during a game and had similar overall jump counts in practice to attacking positions. Middle blockers had a greater number of repeated high-intensity efforts for both games and practice compared with other positions
García-de-Alcaraz et al, 2020^11^	Sagittal plane: jump count	The middle blockers showed the greatest jump count regardless of the season period, while the setters performed the smallest. Similar jump count was found in opposites and outside hitters
Ness et al, 2019^29^	Upper extremity arm swing	Monitoring upper extremity performance changes and swing count volume may have implications for injury prevention
Maciel Rabello et al., 2019^27^	Sagittal plane: jump count	At the dominant side, higher cumulative weekly jump count was related to a significant decrease of echo type I percentage
Lima et al, 2019^22^	Sagittal plane: jump count and jump height	Setters made a significantly greater number of jumps than middle blockers and outside hitters, with similar height. However, middle blockers and outside hitters accumulated their jumps in specific moments (frontcourt)
Skazalski et al, 2018^42^	Sagittal plane: jump count and jump height	Setters performed the greatest number of jumps; opposites jumped higher than their teammates

ACWR, acute:chronic workload ratio; MD-1, training session 1 day before the match; MD-2, training session 2 days before the match.

Altundag et al^1^ and Pawlik and Mroczek^32^ expanded their measurement to the frontal and transverse planes, exploring variables like total distance, accelerations, decelerations, metabolic load, speed, and player load. These variables demonstrated a strong correlation between training loads and competition performance.

Ness et al^29^ took a unique approach by focusing on upper extremity arm swing, suggesting that monitoring changes in this metric may have implications for injury prevention.

## Discussion

### Summary of Evidence

The primary objective of this scoping review was to provide a comprehensive overview of the measurement of ETL in volleyball and identify any gaps in current knowledge. As evidenced from the 18 studies reviewed, research on ETL measurement has focused predominantly on sagittal plane movements, particularly involving jumps. While the importance of jumps in volleyball is undeniable, the emphasis on this single aspect leaves other crucial dimensions underexplored, potentially compromising the development of holistic training and injury prevention strategies.

Jump-related metrics, such as jump count, height, and load, were used consistently across the studies to assess the players’ external load. Lima et al^25^ found that a higher number of jumps corresponded to greater jump height, indicating a potential strain on the player’s physical performance. Similarly, Taylor et al^43^ linked fewer jumps and larger variations in ETLs to an increased risk of injuries. In contrast, Piatti et al^33^ identified middle blockers as having the highest jump load, despite not jumping as frequently as setters, which points to the position-specific demands of the sport.

While the focus on jumping metrics in volleyball research is pervasive due to the sport’s inherent vertical demands, other non-jumping variables also play a pivotal role in defining the ETL for players. Essential parameters such as total distance covered, changes in direction, accelerations, decelerations, metabolic load, speed, player load, and upper extremity movement metrics, particularly the arm swing characteristics, are indispensable in offering a holistic perspective.^1,32^ Especially in positions like libero, which involve minimal jumps, these metrics become paramount. In addition, factors such as kinetic energy in the sagittal plane, lateral movements in the frontal plane, and complex rotational movements in the transverse plane, associated with different player positions, significantly contribute to the overall external load. Hence, to craft a complete narrative of a player’s exertion and the sport’s physical demands, it is imperative to incorporate and emphasize these nonjumping metrics.

Importantly, the position of the libero was notably absent in most studies, perhaps because of the traditional focus on sagittal plane movements. Given that liberos are less involved in jumping activities and have unique role-specific movements,^12^ this omission suggests a substantial area of underrepresentation. In addition, setters often perform complex rotational movements in the transverse plane when setting the ball, and these movements are vital to their performance yet often overlooked in research.^31^ Moreover, the dynamic lateral movements, characteristic of outside hitters when they approach for a spike or adjust their position for blocking, also constitute significant load in the frontal plane.^41^ Understanding these movements in detail could provide unique insights into the physical demands of these positions and could inform training and injury prevention strategies accordingly. By isolating the unique physical demands of each position, coaches can design training programs that focus on strengthening the relevant muscles, enhancing the necessary energy systems, and honing the requisite coordination patterns. This targeted approach ensures that athletes’ training time is spent more efficiently, developing the exact strengths and skills that will be most frequently used in matches. This could be a game-changer, allowing the development of position-specific programs that cater to the unique demands of each role, potentially enhancing performance and reducing injury risk.

Notably, Ness et al^29^ introduced the upper extremity arm swing as a significant metric, suggesting that changes in this measure could have implications for injury prevention. Understanding upper extremity movement is indeed critical when considering the ETL of volleyball players, particularly given the sport’s heavy reliance on upper body actions such as serving, spiking, and blocking.^38,41^ Technologies such as wearable IMUs, which can accurately capture 3-dimensional movement data, are well-suited for this purpose. Metrics derived from such data, including arm swing velocity, frequency, and range of motion, can provide nuanced insight into the demands placed on the upper body during different phases of play. While these measures have broad applicability across all positions due to the integral role of upper body movements in volleyball, they may be particularly valuable for outside hitters, opposites, and middle blockers, who frequently perform high-intensity, upper body-dominant actions.^41^

While these studies have made valuable contributions to our understanding of ETL in volleyball, the concentration of research on sagittal plane measurements, particularly those related to jumping, indicates a substantial gap in current knowledge. The complexity and fast-paced nature of volleyball necessitate a broader examination of ETL that encompasses multiple planes of movement, various player positions, and different levels of play. Such a holistic approach would not only enhance our understanding of volleyball’s physical demands but also aid in the development of tailored training programs and injury prevention strategies.

As previously highlighted, a thorough understanding of ETL is vital for coaches, athletes, and stakeholders involved in the sport, informing the creation of individualized training regimes and injury prevention strategies. Policymakers and healthcare providers could also benefit from this comprehensive overview, allowing them to make informed decisions about volleyball training, competition policies, and player health and safety protocols.

### Limitations

Despite efforts to be comprehensive, only English language articles were included, potentially excluding significant research conducted in other languages. Furthermore, this review did not assess the methodological quality of the included studies, as is typical in a scoping review.^46^ Although this approach allows for the inclusion of a wide range of studies to provide a broad overview of the field, it also means that the strength of the evidence presented cannot be definitively appraised. Consequently, readers should interpret these findings with an understanding of this inherent limitation.

While our primary focus was on examining the methodologies used for measuring ETL in volleyball, we recognize certain intrinsic factors that can influence workload. These factors include potential differences in workload by sex, level of play, and specific type of play (eg, indoor vs beach). The present scoping review did not delve into these variations, as the current objective was more methodologically oriented.

## Conclusion

A key finding is that the majority of the studies focused on sagittal plane measurements, particularly those related to jumping, due to the critical role these actions play in the sport. However, this reveals a gap in the research where other planes and variables, including the frontal and transverse planes, and factors such as kinetic energy, changes of direction, and repeated high-intensity efforts, are often overlooked. Moreover, while it is clear that the measurement of ETL is well-explored for some positions (eg, setters, middle blockers, and outside hitters), there is a conspicuous lack of focus on the liberos. This further highlights the need for a more holistic approach to studying external load in volleyball, taking into account the unique characteristics and demands of different player positions.

In terms of technologies used for monitoring ETL, most of the studies relied on IMUs as the primary tools of data collection. This reflects a predominant methodology in external load measurement in volleyball. The predominance of IMU technology suggests a degree of standardization in the tools used for external load measurement in volleyball, which aids in comparison and synthesis of findings across different studies. Yet, given the multidirectional nature of volleyball,^44^ it is vital to employ technologies capable of capturing movements across all 3 planes - sagittal, frontal, and transverse. Examples of such technologies include sophisticated IMUs and LPSs, which provide comprehensive movement data. When analyzing this data, it is crucial to consider metrics that capture total movement load. While metrics such as PlayerLoad, which is calculated as the square root of the sum of the squared instantaneous rate of change in acceleration in all 3 planes, offer insights into a player’s exertion, one must be cautious.^30^ PlayerLoad and similar metrics are brand-specific, and their algorithms can differ significantly across brands. This can make comparisons challenging and, at times, unfeasible. Therefore, while such metrics provide a convenient tool for coaches, there is a pressing need for more standardized and universally applicable measures to ensure consistency in assessing players’ exertion, taking into account not only the volume of movement but also its intensity and directionality.

For a more granular analysis, coaches can look at specific metrics in each plane. In the sagittal plane, jump count, jump height, and kinetic energy are key variables often measured in volleyball. In the frontal and transverse planes, variables such as total distance covered, changes of direction, accelerations, and decelerations can provide valuable insights into the player’s lateral and rotational movements. It is important to note that while these metrics can be valuable, the accuracy of traditional GPS and accelerometery devices can be limited in indoor settings like volleyball courts. The challenges posed by indoor environments, such as interference and lack of direct GPS signal, can impact data quality. LPS have emerged as potential solutions for indoor tracking, but they too have their set of challenges. Despite these current limitations, these plane-specific metrics can still offer valuable insights and help coaches design position-specific training programs and injury prevention strategies, tailored to the unique demands of each role on the team.

With regard to the existing literature, this review’s findings align with the understanding that volleyball is a sport with high vertical load.^25^ However, the importance of expanding the perspective beyond sagittal plane metrics and considering other positional roles, like libero, are novel insights. From a practice perspective, these findings underscore the need for a multidimensional approach to load monitoring in volleyball, one that accounts for the different planes of movement, role-specific demands, and the overall match and training context. This has potential implications for the design of training programs, with more nuanced and position-specific strategies likely to be more effective in enhancing performance and minimizing injury risk.

As for future research, there is a clear need for more comprehensive studies examining the external load across different player positions, especially the under-researched libero role, and including metrics beyond the sagittal plane. Such research could enable the development of a more complete picture of the physical demands of volleyball, paving the way for more effective, position-specific training programs. Also, further investigation into the different technologies used for load measurement, and perhaps the development of a standardized set of tools, could be beneficial. Furthermore, upper body metrics could be instrumental in individualizing training programs and developing targeted injury prevention strategies.^39^ For instance, identifying a player with a particularly high arm swing velocity might suggest a need for increased focus on shoulder strength and stability in their training regimen. Alternatively, observing changes in arm swing characteristics over the course of a season could provide early indicators of overuse or fatigue, informing timely interventions to prevent injury.^45^ Therefore, the inclusion of upper body movement metrics in future ETL research represents an important avenue for enhancing our understanding of volleyball’s diverse physical demands and tailoring training interventions accordingly.

In conclusion, while this scoping review has provided valuable insights into the measurement of ETL in volleyball, it also highlights significant gaps in the current body of knowledge. Addressing these gaps could hold the key to developing more effective training methods and injury prevention strategies, ultimately elevating the sport.

## Supplemental Material

sj-docx-1-sph-10.1177_19417381241237738 – Supplemental material for Beyond the Jump: A Scoping Review of External Training Load Metrics in Volleyball Supplemental material, sj-docx-1-sph-10.1177_19417381241237738 for Beyond the Jump: A Scoping Review of External Training Load Metrics in Volleyball by André Rebelo, João R. Pereira, Fábio Y. Nakamura and João Valente-dos-Santos in Sports Health
